# Monitoring immunological COVID-19 vaccine clinical testing across the CEPI Centralized Laboratory Network

**DOI:** 10.3389/fimmu.2025.1569251

**Published:** 2025-04-03

**Authors:** Lauren M. Schwartz, Jose Vila-Belda, Jerome Carless, Sadish Dhakal, Koen Hostyn, Trina Gorman, Deborah Ogbeni, Gathoni Kamuyu, Mark Manak, Valentina Bernasconi, Ali Azizi

**Affiliations:** ^1^ Gorman Consulting, Edmonds, WA, United States; ^2^ Coalition for Epidemic Preparedness Innovations (CEPI), London, United Kingdom; ^3^ Turesol Consulting, King of Prussia, PA, United States; ^4^ Coalition for Epidemic Preparedness Innovations (CEPI), Oslo, Norway; ^5^ Coalition for Epidemic Preparedness Innovations (CEPI), Washington, DC, United States

**Keywords:** vaccine results, data management, quality, trial monitoring, database

## Abstract

The CEPI-Centralized Laboratory Network (CLN) has significantly contributed to the development of several approved SARS-CoV-2 vaccines by conducting over 70,000 clinical samples for testing from various vaccine developers. A centralized data management system was developed to track, review, store and share immunological clinical results generated from sample testing. The data system ensures the completeness and accuracy of submitted results and checks the set criteria in controls for each assay. Each testing facility within the network submits their results to a secure storage system using report forms with embedded data quality checks. Upon submission, a statistical program runs additional checks to identify errors in completeness and uniqueness. Any discrepancies or errors are shared with the testing facility to rectify. Reports are further reviewed by CEPI-CLN experts before releasing to the vaccine developer. Study results are then consolidated into an internal relational database management system, enabling CEPI to analyze the data through an interactive dashboard that visualizes control trends and sample results across all studies. This analysis facilitates the harmonization of immunological data and helps to inform CEPI’s programmatic and strategic decision making. Given the success of this approach with SARS-CoV-2 vaccines, the system will be adopted for new pathogens and assay types currently under development at CEPI-CLN.

## Background

As of May 2024, the CEPI-Centralized Laboratory Network (CLN) has significantly contributed to the development of several approved SARS-CoV-2 vaccines by conducting over 120,000 assay runs (over 70,000 clinical samples) for testing from various vaccine developers worldwide (1–4). In summary six SARS-CoV-2 immunological assays have been developed, validated or qualified, and transferred to the network using the same materials, key reagents, and protocols: three binding assays (S-, RBD, and N-ELISA), a microneutralization assay (MNA), a pseudotyped virus-based neutralization assay (PNA), and an IFN-γ T-cell ELISpot assay. Inter-lab studies using replicate assays, as well as revalidation in receiving facilities, have shown that results are highly reproducible, allowing for direct comparison of different vaccines throughout the network (2). Reliability of clinical sample testing is assured through the implementation of an internal centralized system designed to store sensitive and proprietary data and perform data quality checks on the immunological clinical results, ensuring the integrity and consistency of data collected. Additionally, the system enables trend analysis of reference standards and controls, generated by the Medicines and Healthcare Products Regulatory Agency (MHRA, formerly NIBSC), allowing CEPI-CLN to harmonize results across laboratories and to identify any potential issues or anomalies in the data that indicate of loss of consistency between facilities. This centralized system plays a crucial role in maintaining the quality and integrity of clinical sample testing processes.

## Data pipeline

**Figure 1:**
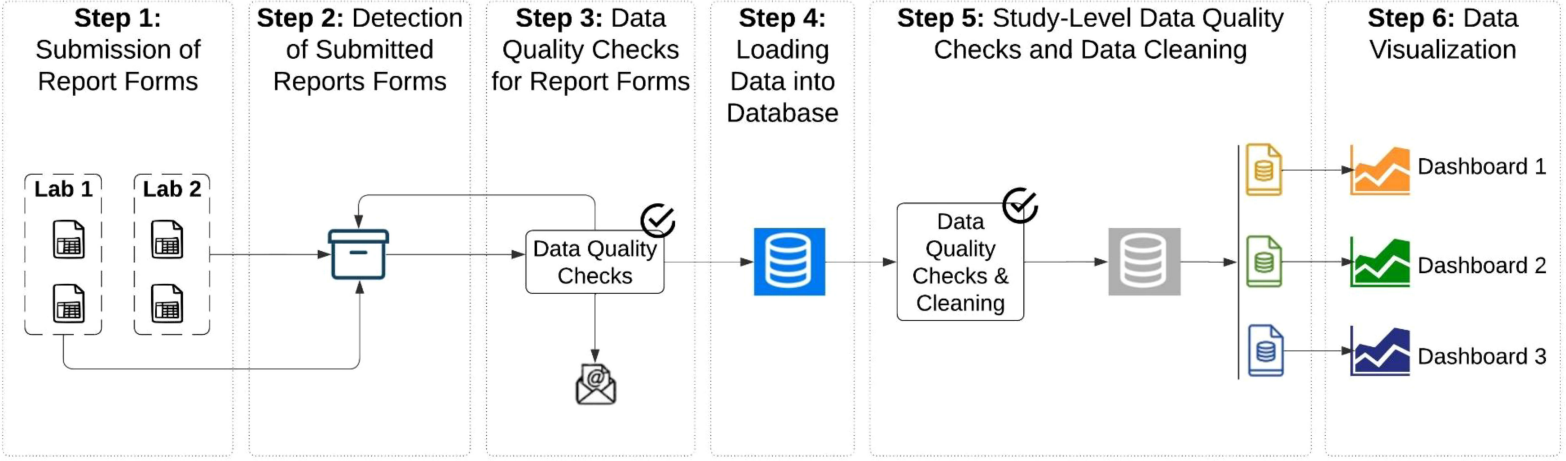
Design of the data pipeline from report submission to visualization. Image shows the different steps of the data pipeline including labs submitting report forms (Step 1), detection of report forms in the centralized storage repository (Step 2), automated data quality checks on the report forms (Step 3), loading (Step 4) and further cleaning of the data in the database (Step 5) and finally visualizations in dashboards (Step 6).

The automated process by which submitted clinical data is checked for consistency, consolidated, cleaned, and stored in a database to enable analysis, is called the data pipeline (**Figure 1**). All steps in the data pipeline were developed by CEPI-CLN and for internal use only. The data pipeline is managed by Apache Airflow (5), an open-source platform that initiates and tracks each dependent step. Further, isolated and encrypted environments on both Amazon Web Services (AWS) and Heroku are used to house Apache Airflow and the database, respectively. Compliance with international data privacy laws (General Data Protection Regulation [GDPR]) (6) and ISO 27001 is achieved through multiple technical and organizational measures that have been deployed throughout the data pipeline including: pseudonymized specimen identifiers, regulated and restricted data access to all software and database storage systems through the principle of least privilege, weekly database and systems backups, and the use of isolated and encrypted cloud environments. The data pipeline was first built within a testing space to ensure a valid and secure pipeline before moving to a production space where regular audits and vulnerability assessments are conducted.

### Step 1: Submission of report forms

Testing facilities within the network have quality management systems in place, employing quality control procedures throughout the analytical process. From sample receipt through to reporting, facilities are responsible for assuring the integrity of the results they generate.

Assay results are entered into a standardized report form at each facility. All facilities participate in virtual training sessions to review consistent data entry protocols. Each report may contain all or a subset of sample testing results for each clinical study with only information relevant for immunologic testing including sample and participant unique identifiers, study time point, data of collection, plate control identifier, and assay result. Additional information collected during the course of a clinical trial, such as participant-level demographic variables, is not shared with the facilities. The report form contains data validation rules, drop down menus, formulas, and conditional formatting (**Table 1**) as a first step in ensuring the completeness and consistency of results. For example, cells change color if any information related to a test sample or plate control is missing. This allows facility staff and reviewers to scan the report to identify missing critical data before submission. Additionally, to complement facility quality control procedures, warnings appear when plate control values fall outside of pre-defined acceptance ranges, signaling samples that need to be retested. The report form is locked, and password protected so that the facility staff cannot accidentally change the embedded functionality. Within each facility, all report forms are approved by a quality control manager or designated expert before being uploaded onto a central encrypted file storage system that is compliant with GDPR (6).

**Table 1:** Data quality checks conducted throughout the data pipeline.

Step	Data Quality Check	Method
Data Collection (Report Form) – Step 1	For key variables (lab name, report status, study ID) only selected list possible	Excel dropdown menu
	Consistent dates across reports and labs	Data format requirement in excel
	Non-missing data on key variables	Color conditioning so that cells with missing data turn orange
	Plate control acceptance criteria calculated correctly according to reagent lot	Excel formula to calculate if controls fall between acceptance criteria, based on lot number
Report-Level Data Quality Checks (Stata Program) – Step 3	For key variables (lab name, report status, study ID) only selected list possible	Program checks for selected known list for each variable
	Consistent dates across reports and labs	Program checks for correct format
	Non-missing data on key variables	Program checks all required fields are filled in
	Unique laboratory specimen IDs and client sample IDs	Program checks for duplicate values by ID and assay date in one report
	Consistent time points	Program checks for consistent time point within a report (Day v Month)
	All accepted sample results come from accepted plates	Program checks that each sample aligns with a run, plate, and assay date from an accepted plate control
	Plate control acceptance criteria calculated correctly according to reagent lot	Program re-calculates formula to ensure users have no user error
Study -Level Data Quality Checks (Database) – Step 5	Unique laboratory specimen IDs and client sample IDs	Program checks for duplicate values by ID and assay date across all reports within a study
	Consistent time points	Program checks for consistent time point across all reports within a study (Day v Month)

### Step 2: Detection of submitted report forms

Following data submission, sample and control immunological results enter CEPI’s automated data pipeline, where each subsequent step is initiated by Apache Airflow (5). The file storage system is automatically checked every hour to detect if new report forms have been submitted. Once a new report form is detected, a python program downloads the file to a temporary directory, triggering a series of automated data quality checks, as described in Step 3.

### Step 3: Data quality checks for report forms

A statistical program designed in Stata is run to ensure each report form retains embedded functionality and calculations, is complete, and passes a series of other quality checks, such as that dates are valid and that plate control acceptance criteria, based on the reagent lot, is calculated correctly (**Table 1**). Results of these checks are saved in a spreadsheet that is uploaded to the central file storage system. An email is sent to relevant CEPI-CLN staff informing them of the result of these checks. The CEPI-CLN team reviews the spreadsheet, identifying and communicating any data quality errors to the facility if needed. Subsequently, the facility may submit a revised report form, which supersedes the previous version, thereby avoiding duplicates in the central file storage system. Report forms approved by CEPI are shared with vaccine developers through the central file storage system. Interpretation of clinical testing results and their relevance to efficacy of the vaccine are the responsibility of the vaccine developer.

### Step 4: Loading data into the database

All rows from the submitted report form with complete sample and plate control data are bulk inserted into a single relational PostgreSQL database. The database is located in a dedicated and isolated environment designed for storing sensitive data. PostgresSQL is inherently ACID compliant. Specifically, the psycopg2 package (7, 8) handles Atomicity and Isolation by managing transactions using connection objects, commits, and rollbacks. Consistency is maintained by enforcing unique constraints across all tables in the database. Durability is achieved through a combination of postgres’ internal Write-Ahead Logging (9) and hourly backup snapshots managed by Heroku (10).

Data is first moved to staging tables which contain all pre-processed data. This provides a historical snapshot of the clinical trial data across each batch. Data are loaded into separate tables for clinical sample results, plate control results, and data quality check summaries. Key identifiers are maintained across all tables and include facility name, assay type, report name, study ID and load date. To maintain idempotency, records matching the bulk insert load date are removed before each insert, ensuring no unexpected duplication.

### Step 5: Study-level data quality checks and data cleaning

A second round of data quality checks are performed within the database across all reports submitted for each study, to ensure all key indicators (sample identifiers, assay type, facility name, study ID, dates) are complete and properly recorded and time points are consistent and valid (**Table 1**). Discrepant results are again reviewed by the CEPI team and, if needed, the facility resubmits revised reports to correct issues. The quality checks are run automatically as each new report is submitted, but it may take weeks until all samples are tested and for final data quality checks to be performed. In the final production tables, version control for revised report forms is maintained through tracking the submission time of each report form and upsert queries ensure that only unique data are inserted. Various data cleaning processes are performed including calculating international standard unit conversions and aligning study visit time point variables across studies (e.g. ‘day1’ and ‘D01’ cleaned to ‘Day 1’). Materialized views are created from the final production tables that aggregate, combine, or reshape data as needed for the visualizations.

### Step 6: Data visualization

**Figure 2:**
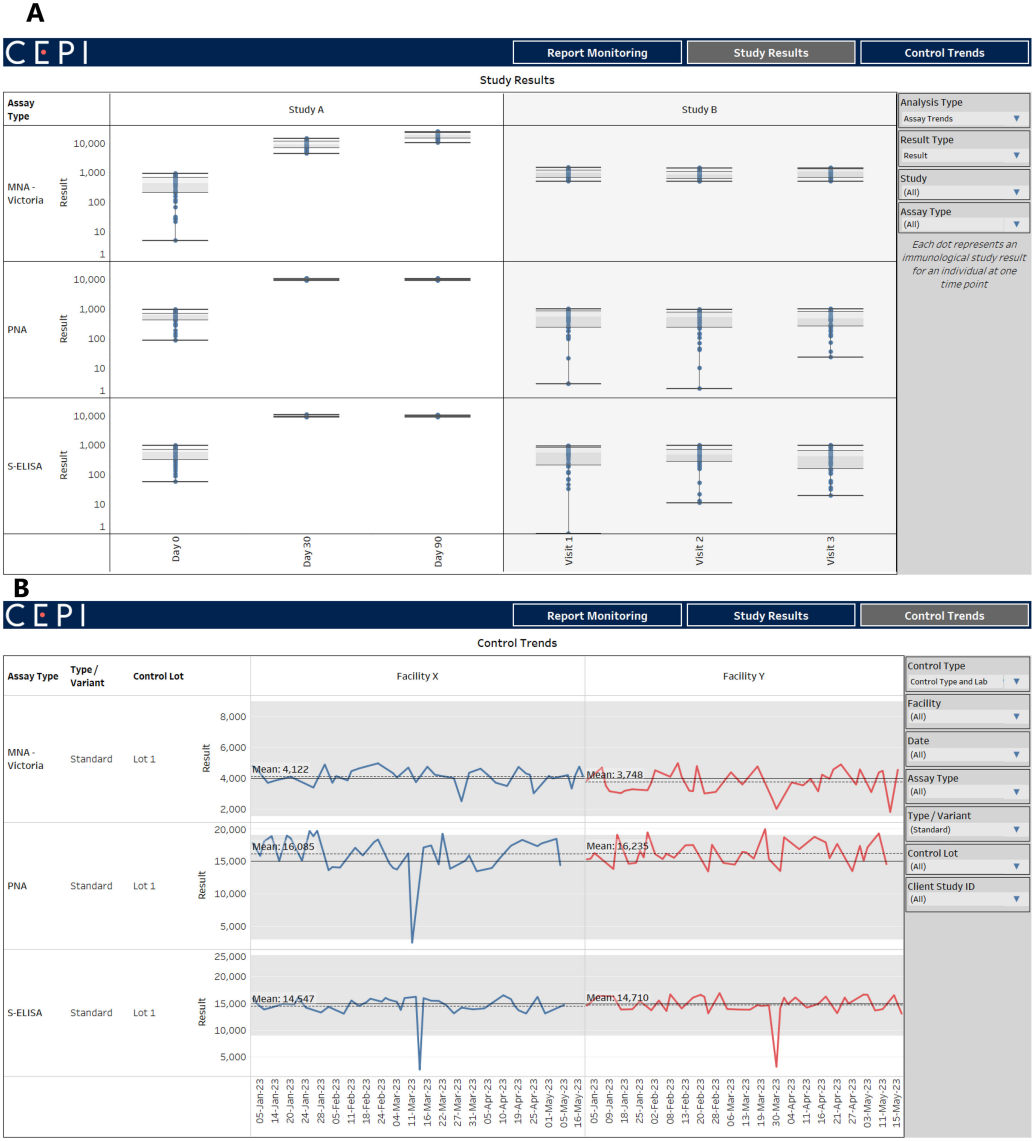
Tableau dashboard with with assay results and control trends. **(A)** shows example clinical trials results on a dashboard connected to CEPI-CLN database. **(B)** shows example control results which can be used to track trends by facility and across time to ensure consistency and identify any possible quality issues.

Using an encrypted connection to the materialized views in the database, an interactive dashboard of data visualizations enables CEPI to continually analyze and monitor submitted results. The dashboard visualizes aspects such as 1) the number and status of reports that have been submitted to support scheduling and inventory control, 2) trends in immunological results by assay and study (**Figure 2A**), and 3) plate control trends by assay, lot, and lab over time (**Figure 2B**). As of May 2024, over 300 reports have been uploaded and quality checked since the start of the data pipeline in 2022. Importantly, control results are used to track trends by facility and across time to ensure consistency and identify any possible quality issues.

## Discussion

The CEPI-CLN currently includes 18 facilities across the world. This network relies on a series of processes to ensure the consistency, completeness, and reliability of vaccine test sample results across all facilities. These processes include standardized and harmonized assay procedures, regular proficiency testing after the post-technology transfer, data quality checks, and ongoing communication and collaboration among the network. By implementing these rigorous processes, the CEPI-CLN aims to maintain high standards of quality assurance and control, ultimately contributing to the development of safe and effective vaccines against emerging infectious diseases. The data quality checks and ongoing analysis provide additional confidence in the data, which is both shared with vaccine developers and used to inform CEPI’s programmatic and strategic decisions. Using the resulting data and visualizations, CEPI can facilitate rapid evaluation and dissemination of the most effective vaccine candidates. Additionally, CEPI can also obtain a better understanding of aspects such as the correlation and duration of protection across multiple SARS-CoV-2 vaccine clinical trials, as well as identifying which vaccine platforms require support towards licensure. Given the success of this approach with COVID-19 vaccines, the system is currently being adopted for new pathogens and assay types currently under development at CEPI-CLN (11). The database may also start to leverage machine learning or Artificial Intelligence (AI) tools to supplement quality control systems. Since 2023, CEPI has made significant investments and partnered with several private companies and recognized academic institutions to incorporate AI-driven tools in various areas to support CEPI’s 100 Days Mission: to quickly make safe and effective vaccines against any viral pandemic threat. Additionally, we plan to incorporate study-level demographic information in the database to support high-level analyses related to vaccine response in different populations. In summary, these processes not only maintain high-quality standards but also strengthen global preparedness, reinforcing CEPI’s commitment to equitable access to vaccines against rare pathogens.

## Data Availability

The datasets presented in this article are not readily available because of privacy concerns. Requests to access the datasets should be directed to author AA, ali.azizi@cepi.net.

## References

[1] Azizi A Manak M Bernasconi V. The CEPI centralized laboratory network for COVID-19 will help prepare for future outbreaks. Nat Med. (2023) 29:2684–5. doi: 10.1038/s41591-023-02534-x 37726389

[2] Manak M Gagnon L Phay-Tran S Levesque-Damphousse P Fabie A Daugan M. Standardised quantitative assays for anti-SARS-CoV-2 immune response used in vaccine clinical trials by the CEPI Centralized Laboratory Network: a qualification analysis. Lancet Microbe. (2024) 5:e216–25. doi: 10.1016/S2666-5247(23)00324-5 38278167

[3] Azizi A Bernasconi V. Unifying global efforts by CEPI’s centralized laboratory network. Front Immunol. (2024) 15:1404309. doi: 10.3389/fimmu.2024.1404309 38665918 PMC11043525

[4] Azizi A Kamuyu G Ogbeni D Levesque-Damphousse P Knott D Gagnon L. Driving consistency: CEPI-Centralized Laboratory Network’s conversion factor initiative for SARS-CoV-2 clinical assays used for efficacy assessment of COVID vaccines. Hum Vaccines Immunother. (2024) 20:2344249. doi: 10.1080/21645515.2024.2344249 PMC11085944 38708549

[5] Apache Airflow. Home (2024). Available online at: https://airflow.apache.org/ (Accessed June 24, 2024).

[6] GDPR.eu. GDPR compliance checklist (2024). Available online at: https://gdpr.eu/checklist/ (Accessed June 24, 2024).

[7] The connection class — Psycopg 2.9.10 documentation (2024). Available online at: https://www.psycopg.org/docs/connection.html (Accessed June 24, 2024).

[8] Basic module usage — Psycopg 2.9.10 documentation (2024). Available online at: https://www.psycopg.org/docs/usage.html (Accessed June 24, 2024).

[9] PostgreSQL Documentation. 28.3. Write-Ahead Logging (WAL) (2024). Available online at: https://www.postgresql.org/docs/17/wal-intro.html (Accessed June 24, 2024).

[10] Heroku PGBackups | Heroku Dev Center . Available online at: https://devcenter.heroku.com/articles/heroku-postgres-backups (Accessed June 24, 2024).

[11] Azizi A Rose K Kamuyu G Ogbeni D Bernasconi V. Preparedness and priority research to tackle the mpox outbreak response. Nat Med. (2025) 31:14–5. doi: 10.1038/s41591-024-03367-y 39753960

